# Cytocompatibility and Wound Healing Activity of Chitosan Thiocolchicoside Lauric Acid Nanogel in Human Gingival Fibroblast Cells

**DOI:** 10.7759/cureus.43727

**Published:** 2023-08-18

**Authors:** Ameena M, Meignana Arumugham I, Karthikeyan Ramalingam, Rajeshkumar S, Elumalai Perumal

**Affiliations:** 1 Oral Pathology and Microbiology, Saveetha Dental College and Hospitals, Saveetha Institute of Medical and Technical Sciences, Saveetha University, Chennai, IND; 2 Oral Pathology, Azeezia College of Dental Sciences and Research, Kollam, IND; 3 Public Health Dentistry, Saveetha Dental College and Hospitals, Saveetha Institute of Medical and Technical Sciences, Saveetha University, Chennai, IND; 4 Pharmacology, Saveetha Dental College and Hospitals, Saveetha Institute of Medical and Technical Sciences, Saveetha University, Chennai, IND

**Keywords:** human gingival fibroblast cells, thiocolchicoside, lauric acid, cytotoxic effect, cytocompatibility, nanogel

## Abstract

Aim: To investigate the cytocompatibility effect and wound healing activity of chitosan thiocolchicoside lauric acid (CTL) nanogel using human gingival fibroblast (hGF) cells.

Materials and methods: hGF cells were established from gingival tissue as per the standard cell isolation protocol. The cytocompatibility effect was assessed using an MTT (3-(4,5-dimethylthiazol-2-yl)-2,5 diphenyl tetrazolium bromide) assay. A scratch wound healing assay was performed to assess the wound-healing potential of CTL nanogel. For the nuclear morphological changes analysis, acridine orange staining was used in gingival fibroblast cells. The stained nuclei were viewed under a fluorescent microscope. ANOVA with posthoc analysis was performed using GraphPad Prism 5 software (Dotmatics, Boston, Massachusetts). The significance level (p-value) was expressed as <0.05.

Results: CTL nanogel did not show any significant cytotoxicity at concentrations 10-80 µl/ml (p<0.05). CTL nanogel at a concentration of 40µl/ml has a cytocompatibility effect on hGF cells and increases cell viability. In vitro scratch wound healing assay resulted in faster wound healing and cell migration with CTL nanogel when compared to the control group.

Conclusion: CTL nanogel has a significant effect on cell proliferation at various concentrations, which suggests its use as a safe and effective drug delivery system.

## Introduction

Wound healing is a very crucial process to revive the integrity of the tissue and its function after injury or trauma [1]. The principal goal in wound healing is the proper closure of the wound, which occurs in four important stages: hemostasis, inflammation, proliferation, and tissue remodeling [2]. There are different factors that affect the rate of wound healing and numerous researches are being carried out in this field to find out newer drugs that accelerate healing of wounds. Scratch wound healing assay is an in vitro analysis that is a reliable, cheap, and faster method and mimics the migration of cells during the in vivo healing of wounds [3].

Nanogel is a polymer-based hydrogel particle that is cross-linked at a sub-micron level. It can have a three-dimensional structure with crosslinked polymers containing hydrophilic or amphiphilic macromolecular chains that can swell and hold a great amount of water without dissolving while maintaining the structure intact. Nanogels have been an area of interest in recent research in the treatment of various diseases, including autoimmune diseases, neurodegenerative disorders, diabetes, and inflammatory disorders. Additionally, they are helpful for intracellular delivery and can serve as drug delivery systems. By delivering drugs directly to the site of the disease, targeted drug delivery can minimize side effects and increase therapeutic efficacy, while also reducing the dose and frequency of drug administration [4-7].

Lauric acid is a medium-chain saturated fatty acid that is commonly used in the production of soaps and cosmetics. It was subjected to react with sodium hydroxide to produce sodium laurate, which is soap. Recent research has shown that lauric acid exhibits a broad spectrum of antimicrobial activities against enveloped viruses and various bacteria. These properties suggest that it could be useful for protecting against microbial infections and controlling the balance and distribution of bacteria in the human gut microbiota [8]. Lauric acid has also been utilized as a biological control agent [9]. Furthermore, lauric acid has been explored as a potential medicine [10]. In vitro studies have demonstrated that lauric acid induces apoptosis in colon cancer cells due to oxidative stress [11].

Thiocolchicoside is a semi-synthetic colchicine derivative that is widely used as an analgesic and anti-inflammatory drug. It has muscle relaxant properties and is commonly prescribed for orthopedic, traumatic, and rheumatologic disorders. Thiocolchicoside is indicated as an adjuvant drug for the treatment of painful muscle contractures and acute spinal pathology in adults and adolescents over the age of 16 years [12], and has been used as a muscle relaxant for a long time. Its chemical structure contains colchicine, a sugar (ose), and a sulfur-containing radical (thio) [13]. Methods have been developed to assess the bioequivalence of thiocolchicoside as a single component and in fixed-dose combination tablets with lornoxicam [14]. Thiocolchicoside has a potent convulsant activity and is contraindicated for patients with epilepsy [15]. The drug has a selective affinity for the inhibitory gamma-aminobutyric acid and is commonly used to treat acute painful muscle spasms [16].

Chitosan nanoparticles have become an interesting research material in the biomedical world due to their excellent antimicrobial properties, biocompatibility, and biodegradability. The antibacterial activity of chitosan can be explained by mechanisms like intracellular leakage, inducing functional defect, and altered permeability of the membrane, finally resulting in cell death. Chitosan has increased wound healing efficiency, especially when modified with other natural compounds. Polycationic chitosan nanoparticles have increased drug-loading capacity, increased surface area-to-volume ratio, high functionalization, and excellent antibacterial potential compared to chitosan [17].

In this present study, thiocolchicoside and the lauric acid formulation were transformed into a nanogel using chitosan polymer (we are calling it Chitosan Thiocolchicoside Lauric Acid (CTL) Nanogel) and tested for its cytotoxic effect and wound healing potential using human gingival fibroblast (hGF) cells.

## Materials and methods

This study was carried out at Saveetha Dental College and Hospitals, Chennai, India.

Preparation of CTL nanogel

**Figure 1:**
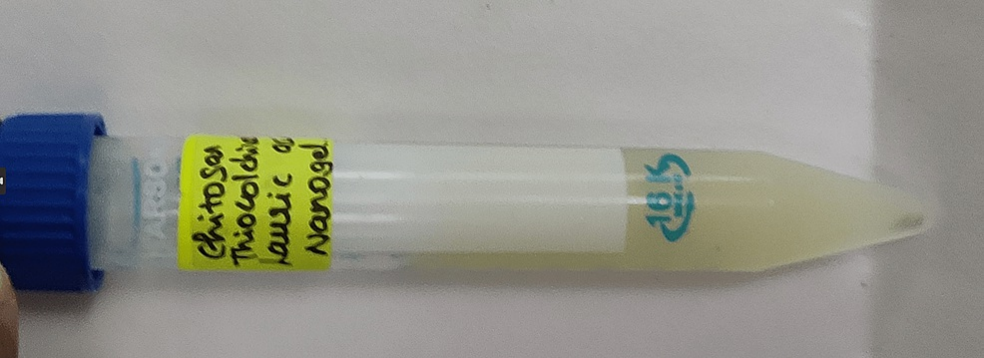
Chitosan thiocolichoside lauric acid nanogel formulated in our study

For the preparation of the components for the nanogel formulation, 0.5 g of lauric acid was dissolved in 10 mL of ethyl alcohol (lauric acid solution), and 50 mg of thiocolchicoside was dissolved in 10 mL of distilled water (thiocolchicoside solution). The nanogel was prepared by combining the two solutions; 5mL of the lauric acid solution and 5mL of the thiocolchicoside solution were mixed together using a magnetic stirrer, and the mixture was stirred continuously for 24 hours. After the stirring process, the resulting mixed solution was added to 10mL of medium molecular-weight chitosan (Figure 1).

We used the following chemicals for this study: Dulbecco's Modified Eagle Medium/Nutrient Mixture F-12 (Gibco™ DMEM F-12; Thermo Fisher Scientific Inc., Waltham, Massachusetts, United States), antibiotics (streptomycin, penicillin), trypsin-EDTA (Gibco™ Trypsin-EDTA; Thermo Fisher Scientific Inc.), phosphate buffer saline (Gibco™ PBS; Thermo Fisher Scientific Inc.), fetal bovine serum (FBS) (Gibco Fetal Bovine Serum FBS; Thermo Fisher Scientific Inc.). MTT (3-(4, 5-dimethylthiazol-2-yl)-2, 5-diphenyl tetrazolium bromide) reagent (Sigma-Aldrich; St. Louis, Missouri, United States) and dimethyl sulfoxide (DMSO) (Sigma Aldrich). The other reagents used for this study were analytical grade.

Establishment of hGF primary cells

The gingival tissue collection method was approved by the Institutional Human Ethical Committee of Saveetha Dental College (approval number: IHEC/SDC/PhD/2118/23/195). Gingival tissues were procured from interdental papillae of healthy adolescent patients during the extraction of their premolars during orthodontic therapy. The patients were informed and they signed an approved consent form before tissue collection. Tissues were weighed (20-50 mg) and kept for one to four hours in a sterile saline solution before processing. Before the experiments, all sterilization protocols were followed and tissue processing was done in a biosafety cabinet. To dilute the oral bacterial flora, the human gingival tissues were washed 10 times in PBS. The tissues were sliced into tiny fragments of 1-2 mm^2^ using a surgical blade no.11 on a sterile Petri plate containing the culture media DMEM F12 and Ham's F-12 Nutrient Mix (Thermo Fisher Scientific Inc.) after being washed in PBS. The human gingival tissue was plated onto 25 cm^2^ tissue culture flasks and this was left undisturbed for 48 hours at a temperature of 37°C in a humidified incubator with 5% CO_2_ for 24 hours. The medium was changed every 48 hours until the number of cells was large enough to carry out the experiment.

The cell viability MTT assay

The hGF cells were plated separately in 96 well plates with a concentration of 5×10^3^cells/well in DMEM media with 10% FBSand 1X antibiotic solution in a CO_2_ incubator at 37˚C with 5% CO_2_. A quantity of 100 μL of 1X PBS was used to wash the cells and then the cells were treated with CTL nanogel and incubated in a CO_2_ incubator at 37˚C with 5% CO_2_ for 24 hours. At the end of the treatment period, the medium was aspirated from cells. MTT (0.5 mg/mL in 1X PBS) was prepared and incubated at 37˚C in a CO_2_ incubator for four hours. The MTT-containing medium was discarded after the incubation period from the cells and washed using 100 μL of PBS. The crystals formed were dissolved and thoroughly mixed with 100 μL of DMSO. The formazan dye changes the color to purple-blue and the absorbance was measured at 570 nm using a microplate reader. The percentage cell viability is measured using the given formula: cell viability = (optical density (OD) of treated cells/OD of control cells) × 100.

To study the effect of CTL nanogel on cell morphology, hGF cells (2x10^5^) were plated in six well plates and the cells were treated with and without the nanogel for 24 hours. The cells were washed with PBS after the treatment period and observed in an inverted phase contrast microscope.

Scratch wound healing assay

hGT cells (2×10^5^ cells/well) were plated onto six-well culture plates. The cell monolayer was scratched using a 200 μl tip to develop a wound, was washed using PBS, and images were captured in an inverted microscope (Euromex Microscopen B.V., Arnhem, Netherlands). The cells were treated with CTL nanogel (20 and 40 μg/ml) for 24 hours and the control cells received serum-free culture medium. The wound area images were taken after treatment and the experiments were repeated in triplicate for each treatment group.

Acridine orange staining

The hGF cells (5x10^4^ cells/well) were plated in six well plates for live imaging using acridine orange (Product No. A6014-10G; Sigma Aldrich) staining. The cells were treated with and without CTL nanogel (20 μl/ml and 40 μl/ml) for 24-hour time points. After the incubation period, the cells were washed using PBS and then stained with acridine orange (1 μg/mL). After staining, the cells were washed with PBS twice and the stained cells were examined under an inverted fluorescence microscope.

Statistical analysis

The statistical test performed for this study was a one-way analysis of variance (ANOVA) with posthoc analysis (Tukey's multiple comparison tests) to compare the mean values of cell viability between different groups (control vs. CTL nanogel-treated groups) using GraphPad Prism 5 software (Dotmatics, Boston, Massachusetts, United States). The data were denoted as means ± SD (standard deviation) and the significance level (p-value) was expressed as <0.05. 

## Results

**Figure 2:**
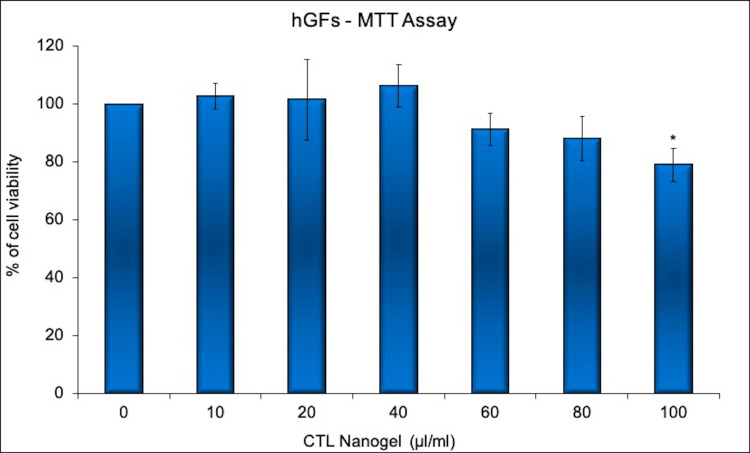
Cell viability assay showing the cytotoxic effects of CTL nanogel on hGF cells Cells were treated with CTL nanogel (0– 100µl/ml) for 24 hours and cell viability was evaluated by MTT assay. Data are shown as means ± SD (n = 3)* compared with the control group, p < 0.05. CTL: chitosan thiocolchicoside lauric acid; MTT: 3-(4,5-dimethylthiazol-2-yl)-2,5 diphenyl tetrazolium bromide; hGF: human gingival fibroblast

The cells were treated with CTL nanogel at varied concentrations (10-100 µl/ml) for 24 hours, and the viability of the cells was analyzed using the MTT assay. The data were presented as means ± standard error (n=3) and the percentage of control and p-value were also provided. The results show that treatment with CTL nanogel at concentrations of 60 µl/ml and above-reduced cell viability compared to the control group. CTL nanogel at 10-40 µl/ml concentration promotes hGFs cell viability. The percentage of cell viability decreased with increasing concentrations of CTL nanogel, indicating a dose-dependent cytotoxic effect. The p-value for the up to 60 µl/ml and 80 µl/ml concentrations of CTL nanogel was not less than 0.05, indicating a statistically non-significant difference when compared to the control group only (Figure 2).

The treatment with CTL nanogel 100 µl/ml concentrations showed significant cytotoxicity to the hGF cells. However, the p-value for this concentration was 0.023, which is less than 0.05, indicating a statistically significant difference compared to the control group. This suggests that treatment with CTL nanogel at a concentration of 40 µl/ml has a cytocompatibility effect on hGF cells and increases cell viability. The percentage of cell viability decreased with increasing concentrations of CTL nanogel, indicating a dose-dependent cytotoxic effect.

**Figure 3:**
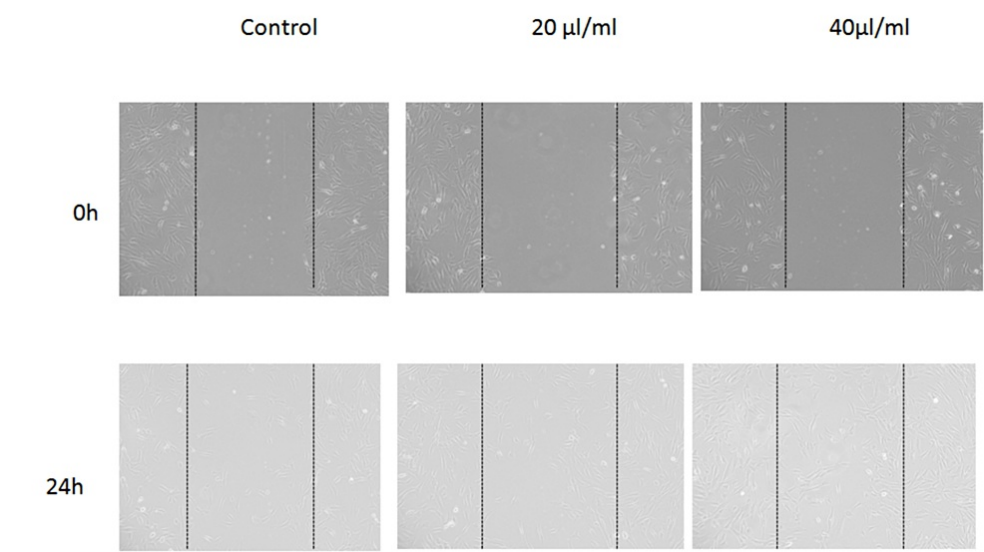
In vitro scratch wound healing assay Human gingival fibroblast cells were injured and cell migration assay with and without treatment (20 μl/ml and 40 μl/ml concentrations) of CTL nanogel was performed. Images were obtained using an inverted phase-contrast microscope. CTL: chitosan thiocolchicoside lauric acid

Based on the in vitro scratch wound healing assay, it appears that treatment with CTL nanogel resulted in faster wound healing and cell migration compared to the control group (Figure 2). Specifically, the images obtained from the assay showed increased closure of the scratch wound in the treatment groups compared to the control group at 24 hours. This effect was observed at both the 20 μl/ml and 40 μl/ml concentrations of CTL nanogel. These results suggest that the CTL nanogel may have the potential for promoting wound healing and cell migration in human gingival fibroblast cells (Figure 3).

**Figure 4:**
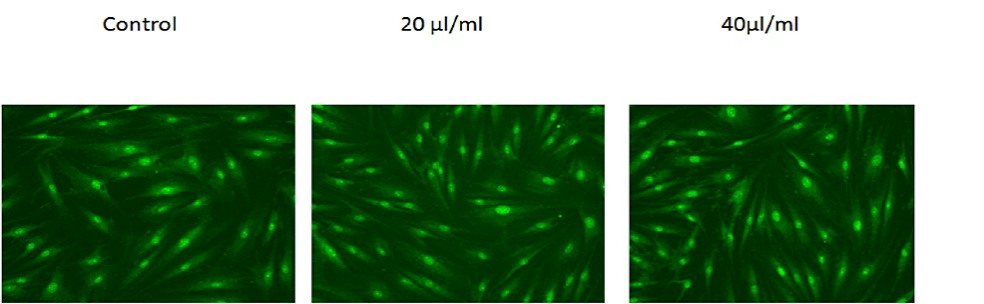
Inverted microscope photomicrographs Human gingival fibroblast cells were treated with 20 μl/ml and 40 μl/ml concentrations of CTL nanogel for 24 hours along with the control group. After this, cells were treated with acridine orange, and images were obtained using an inverted microscope. CTL: chitosan thiocolchicoside lauric acid

Based on the images obtained from the inverted microscope, it appears that treatment with CTL nanogel resulted in morphological changes in hGF cells compared to the control group. Specifically, the cells treated with CTL nanogel at 20 μl/ml and 40 μl/ml concentrations showed a more elongated and spindle-like shape compared to the control group. These changes in cell morphology suggest that the nanogel may be influencing the cytoskeleton of the cells, which can play a role in cell migration and wound healing. Treatment with CTL nanogel resulted in changes in the staining pattern of hGF cells compared to the control group (Figure 4).

Specifically, the cells treated with CTL nanogel at both the 20 μl/ml and 40 μl/ml concentrations showed a brighter and more homogeneous staining pattern with acridine orange compared to the control group. Nucleic acid-selective fluorescent dye, acridine orange, can bind to DNA and RNA, allowing visualization of the nuclei and cytoplasm of cells. These changes in the staining pattern suggest that the nanogel may be influencing the DNA and RNA content of the cells, which could be related to cell proliferation and migration.

## Discussion

Our results suggest that the prepared CTL nanogel may have the potential for modulating cellular processes in hGF cells, which could have implications for the treatment of oral wound healing [18,19]. Previous research has indicated that lauric acid, a saturated fatty acid that is commonly present in coconut oil, possesses antimicrobial and antiviral properties [20,21]. It has been demonstrated that lauric acid has potent bactericidal properties and can be employed as a natural antibiotic against *Propionibacterium* acnes. Furthermore, one study has shown that lauric acid did not have an impact on cell viability in human sebocytes at the concentrations that were tested [22].

In vitro studies have shown that thiocolchicoside does not significantly affect cell viability even at high concentrations of up to 100 µM [23]. Thiocolchicoside is a muscle relaxant that works by selectively binding to the GABA-A receptor and activating the GABA-inhibitory motor pathway. It has been prescribed for the treatment of orthopedic, traumatic, and rheumatologic disorders due to its muscle relaxation, analgesia, and local anesthetic activities [24]. However, there is no research on the cell viability and cell migration property of CTL nanogel on hGF cells. Further studies are needed to investigate the molecular mechanism of this nanogel in promoting hGF cell viability.

Nanogel formulations from naturally occurring products have been used in combating clinical situations like oral mucosal lesions [25] and gingival and periodontal diseases [26-28]. Moreover, nanogel has excellent stability, making it an ideal carrier for drugs with short half-lives. Nanogel's ability to encapsulate both hydrophobic and hydrophilic drugs could be harnessed to invent targeted drug delivery systems, potentially leading to improved patient outcomes and reduced healthcare costs. We have planned to identify the role of our novel nanogel in animal studies and ultimately implement it as an oral aid for patients. Nanoparticles could be used for diagnostic and treatment modalities [25] and also in the development of paper-based biosensors for oral cancer screening [29].

In vitro scratch wound healing assay is a useful in vitro analysis that tests wound healing in which a wound is created on a monolayer of cells, and the cells are treated with a chemical or drug of choice. Images are captured at intervals after treatment to evaluate the migration of cells and cellular interactions [3]. Saporito et al. found cellular proliferation and biocompatibility of nanostructured lipid carriers with eucalyptus and olive oil towards normal human fibroblasts in an in vitro wound healing model. They concluded that the results are due to the presence of oleic acid in olive oil [30].

Limitations

The present study is only an in vitro analysis of our novel CTL nanogel. There is a need for further in vivo analyses to prove the efficacy of the novel CTL nanogel and to explore its role as a potent wound-healing medication.

## Conclusions

The present study evaluated the cytocompatibility of CTL nanogel on normal hGF cell lines. The findings suggest the use of CTL nanogel as a therapeutic agent that could be useful in medical applications. CTL nanogel has several advantages including its good wound healing capacity and biocompatibility. Moreover, it is non-immunogenic and can be easily synthesized and functionalized for a wide range of applications. Acridine orange staining pattern strongly suggests that CTL nanogel affects the DNA and RNA content of the cells, which could be related to cell proliferation and migration. The present study provides a strong foundation for continued investigation of this innovative nanogel as an oral wound healing agent.
